# Phylogenetic and regulatory region analysis of *Wnt5 *genes reveals conservation of a regulatory module with putative implication in pancreas development

**DOI:** 10.1186/1745-6150-5-49

**Published:** 2010-08-04

**Authors:** Maria Kapasa, Stilianos Arhondakis, Sophia Kossida

**Affiliations:** 1 Bioinformatics & Medical Informatics Team, Biomedical Research Foundation of the Academy of Athens, 11527, Athens, Greece; 2 Developmental Biology Laboratory, Biomedical Research Foundation of the Academy of Athens, 11527, Athens, Greece; 3 Department of Pharmacy, School of Health Sciences, University of Patras, GR-26500 Rion-Patras, Greece

## Abstract

**Background:**

*Wnt5 *genes belong to the large *Wnt *family, encoding proteins implicated into several tumorigenic and developmental processes. Phylogenetic analyses showed that *Wnt5 *gene has been duplicated at the divergence time of gnathostomata from agnatha. Interestingly, experimental data for some species indicated that only one of the two *Wnt5 *paralogs participates in the development of the endocrine pancreas. The purpose of this paper is to reexamine the phylogenetic history of the Wnt5 developmental regulators and investigate the functional shift between paralogs through comparative genomics.

**Results:**

In this study, the phylogeny of *Wnt5 *genes was investigated in species belonging to *protostomia *and *deuterostomia*. Furthermore, an *in silico *regulatory region analysis of *Wnt5 *paralogs was conducted, limited to those species with insulin producing cells and pancreas, covering the evolutionary distance from agnatha to gnathostomata. Our results confirmed the *Wnt5 *gene duplication and additionally revealed that this duplication event included also the upstream region. Moreover, within this latter region, a conserved module was detected to which a complex of transcription factors, known to be implicated in embryonic pancreas formation, bind.

**Conclusions:**

Results and observations presented in this study, allow us to conclude that during evolution, the *Wnt5 *gene has been duplicated in early vertebrates, and that some paralogs conserved a module within their regulatory region, functionally related to embryonic development of pancreas. Interestingly, our results allowed advancing a possible explanation on why the *Wnt5 *orthologs do not share the same function during pancreas development. As a final remark, we suggest that an *in silico *comparative analysis of regulatory regions, especially when associated to published experimental data, represents a powerful approach for explaining shift of roles among paralogs.

**Reviewers:**

This article was reviewed by Sarath Janga (nominated by Sarah Teichmann), Ran Kafri (nominated by Yitzhak Pilpel), and Andrey Mironov (nominated by Mikhail Gelfand).

## Background

**Figure 1:**
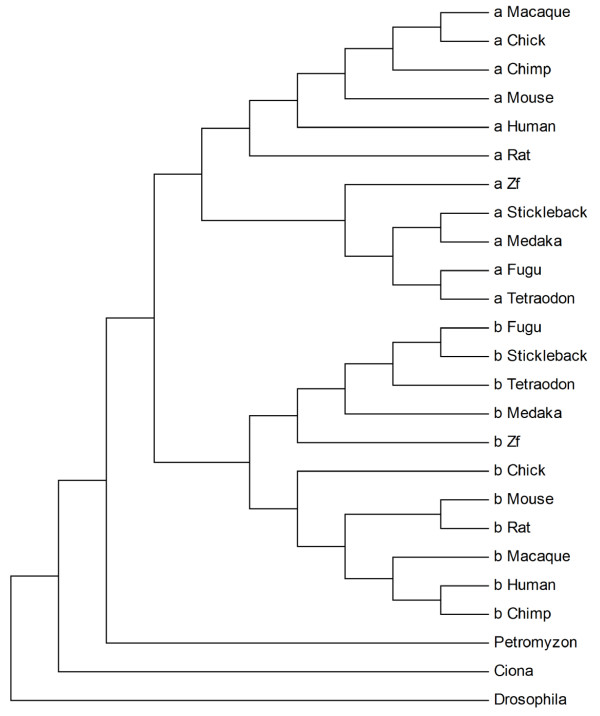
**Phylogenetic tree of Wnt5a and Wnt5b proteins**. Letters a and b, respectively denote the Wnt5a and Wnt5b paralogs.

*Wnt *genes have undergone a rapid structural and functional change in a surprisingly short period of time, <100 million years ago (MYA; 1). In particular, the *Wnt5 *gene has been found to be duplicated in those species arisen in evolution later than the divergence time of jawed vertebrates (gnathostomata**) **from the lineage of agnatha, including hagfish and lampreys, nearly 560 MYA [1,2]; see also Figure 1). Indeed species belonging to *protostomia *invertebrates (*Drosophila melanogaster) deuterostomia *invertebrates *(Ciona intestinalis) *and to the class of agnatha vertebrates (*Petromyzon marinus*), were found to have one *Wnt5 *gene, while gnathostomata vertebrates have two, namely *Wnt5a *and *Wnt5b*. These genes encode growth factors, known to be implicated in several developmental and tumorigenic processes [3,4]. Forsooth, it has been shown that Wnt5a signaling determines the migration of insulin-positive cells during murine pancreatic morphogenesis [5]. Moreover, an abnormal formation of pancreas in early embryos of mice occurred after over-expression of *Wnt5a *[6]. Finally, after induction of the key transcription factor (TF) for endocrine pancreas specification, the Neurogenin3 (NGN3), an altered expression level of *Wnt5a *in murine embryonic pancreas progenitors was detected [7], whereas that of *Wnt5b *remained unaltered [8].

A previous study [5] showed that in some species carrying both *Wnt5 *paralogs only one participates in cell migration events during the endocrine pancreas development. More explicitly, they showed that in early mice embryos *Wnt5a *expression guides the migration of islet cells in order to properly form the endocrine part of pancreas, while in zebrafish embryos, the Wnt5 signalling is required for the proper migration of insulin producing cells during pancreas development [5]. However, previous studies aiming to establish the function of *Wnt5 *genes, observed that, despite the similarity in expression pattern between zebrafish ZfWnt5 and murine Wnt5a during pancreatic development [9-12], the former was found to be characterized by a higher amino acid sequence similarity to that of the mouse Wnt5b [3].

Taking into account the importance of *Wnt5 *genes during development of pancreas, in relation to shifting roles between paralogs, we decided to conduct a phylogenetic and an *in silico *comparative regulatory region analysis of *Wnt5 *genes. Our data confirmed that the duplication of the *Wnt5 *gene, including also its upstream region, has occurred at the divergence time of gnathostomata from agnatha. Additionally, within this region we identified a conserved regulatory module binding *trans *regulators annotated to be related to pancreas development.

## Results and Discussion

### Phylogenetic Analysis

The orthologous protein sequences of *Wnt5a *and *Wnt5b *genes were identified in many species, covering a phylogenetic distance from invertebrate *protostomia *to vertebrate *deuterostomia *(see Materials and Methods). The phylogenetic relationships, as estimated from amino acid sequence similarities, are shown in a cladogram tree in Figure 1. According to this tree, the Wnt5 sequences of Ciona, Drosophila and of *P. marinus *(Lamprey*) *were found at separated branches to those of the other Wnt5a and Wnt5b proteins of the gnathostomata.

**Figure 2:**
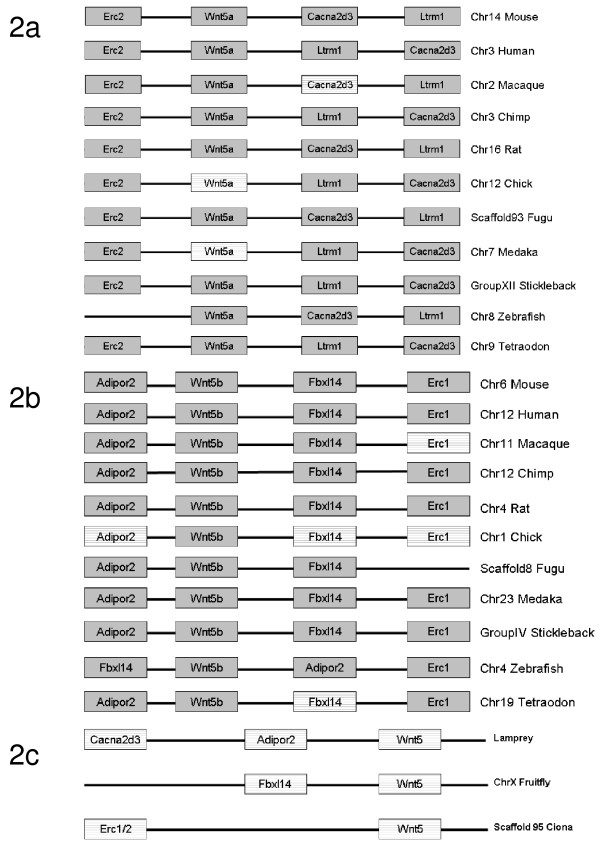
***Wnt5 *syntenies**. The syntenies for the genomic *loci *of *Wnt5a *and *Wnt5b *genes are presented in Figures 2a and 2b respectively, whereas the only genomic *locus *containing a *Wnt5 *gene for Lamprey, Ciona and Fruitfly is shown in Figure 2c. The boxes represent the coding regions of the genes and those with horizontal lines the predicted orthologs of the genes named inside them.

Afterwards, the syntenies for *Wnt5a *and *Wnt5b *genes were investigated (see Figure 2; for details see Materials and Methods). In this figure, the genomic regions surrounding each *Wnt5 *gene were shown to be conserved, and only for some species partial inversions have occurred. Summarizing, the *Wnt5a *gene was surrounded by *Erc2 *and *Cacna2d3*, with *Ltrm1 *being in the genomic neighbourhood of the latter (Figure 2a). On the other hand, *Wnt5b *was surrounded by *Adipor2 *and *Fbxl14*, with *Erc1 *in close proximity of the latter in most species (Figure 2b). Finally, in Figure 2c the syntenic regions for the *Wnt5 *gene of Lamprey (*P. marinus*), Ciona (*C. intestinalis*), and Drosophila (*D. melanogaster*) are presented. For Petromyzon, *Cacna2d3 *and *Adipor2 *genes were identified (Figure 2c), both on the same side of *Wnt5 *gene. It is worth noting that these genes were found to surround *Wnt5a *and *Wnt5b *paralogs in species belonging to gnathostomata. In proximity to *Wnt5 *in Ciona and Fruitfly, *Erc1/2 *and *Fbxl14 *were identified, found also in the syntenic regions of both *Wnt5 *duplicates in gnathostomata. The above findings suggest that a gene duplication event has occurred approximately at the divergence time of gnathostomata, involving the *Wnt5 *gene and its neighbouring genomic region, followed by genomic translocation.

### Regulatory Region Analysis

**Figure 3:**
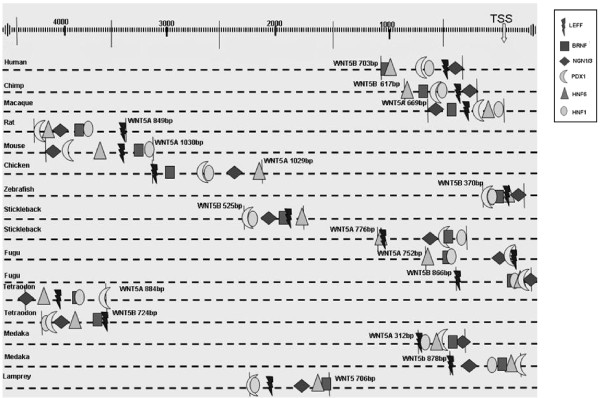
**The regulatory module of *Wnt5***. Schematic representation of the regulatory module for those *Wnt5 *paralogs, in which this cluster of TFBS was identified. The regions demarcating the regulatory module are indicated with vertical lines, and the ruler at the top indicates the distance from the TSS. The TF families are indicated with different symbols: the rhomb is used to depict NEUR binding sites, the triangle HNF6, the circle HNF1, the crescent PDX1, the square BRNF and the thunder LEFF TFBS.

An *in silico *analysis of the regulatory regions of *Wnt5a *and *Wnt5b *genes, was performed only for those species having pancreas and cells producing insulin (see Materials and Methods). Indeed, Ciona and Fruitfly were excluded from the analysis [13-15], while Lamprey, the only living representative of agnatha with known genome, having both pancreas and insulin producing cells [16,17] was maintained. Therefore, the regulatory region analysis was conducted only to species covering the evolutionary distance from agnatha to mammals (Figure 3). Through the application of an extensive filtering method [18] on the selection of transcription factor binding sites (TFBS), a conserved distant regulatory module was identified within the upstream region of some *Wnt5 *genes. Figure 3 shows those *Wnt5 *paralogs for each species in which this module was identified, the TFBS relative positions (in scale), and the absolute distance of the regulatory module from the transcription start site (TSS) of each gene. Strikingly, this conserved regulatory region covers a narrow genomic *locus *containing the binding motifs of 6 TFs belonging to the following families: NEUR (NGN1/3), HNF1, HNF6, BRNF (BRN2, BRN3, BRN4, and BRN5), PDX1 (PDX1 and ISL1), and LEFF (LEF1). The *p-values *of those TFs found to cluster together with NEUR are given in Table 1.

**Table 1:** The *p-values *of the TF families identified in the regulatory module.

TF Family	*p*-value
LEFF	5.1 10^-6^
NEUR	1.2 10^-5^
PDX1	2.4 10^-5^
HNF6	3.7 10^-5^
HNF1	1.1 10^-3^
BRNF	1.5 10^-3^

The first column reports the TF families of those elements identified at the conserved regulatory module and the second one the corresponding *p-values *(probability of random identification of the binding motifs of each element, in the entire submitted sequence).

Despite the variable length of this regulatory module, its changeable distance from the TSS, and the different order where the TFBS are found, the specific region was conserved in all species investigated herein. Indeed, highly conserved transcription regulators are usually expected to be found around and within their target genes as well as at random positions within a genome [19-21] spanning variable distances upstream or downstream of the TSS [22].

It seems that selective pressure during evolution has acted within these genomic sequences, in order to maintain unaltered the binding motifs of the previously referred TFs, known to participate in pancreas development. Explicitly, NGN3 is required for the specification of the cells' subtypes of the endocrine pancreas [7]. On the other hand, PDX1 and ISL1 regulate islet cell development and insulin gene expression, while HNF6 and HNF1 are both transcriptional activators of pancreas-specific genes [23-27]. Moreover, the members of the BRNF family (BRN2, BRN3, BRN4, and BRN5), participate in mammalian embryogenesis by regulating different patterns of gene expression, with BRN4 involved in cell fate determination of cells capable of producing glucagon [28,29]. Finally, LEF1 mediates the effects of the canonical Wnt signaling pathway, on which several organogenic events depend [30].

These TFs are predicted to bind to this regulatory region, during pancreas development, simultaneously or not. Hence, the presence of this under question regulatory module indicates the participation of the respective *Wnt5 *paralog in pancreas differentiation procedures. In order to test the sensitivity of our method we performed exactly the same analysis on the constitutively expressed gene of *b-actin *(see Materials and Methods). Our results showed absence of the regulatory module, with the only exception that of zebrafish *b-actin*, where the same cluster of TFBS to that of *Wnt5 *was identified, although within a region of approximately the double length. The given findings from the above negative control experiment, in relation to the documented implication in pancreas development of the murine *Wnt5a *and the zebrafish *Wnt5b *[3,5], both carrying this cluster of TFBS (Figure 3), further support the functionality of this conserved regulatory module.

**Figure 4:**
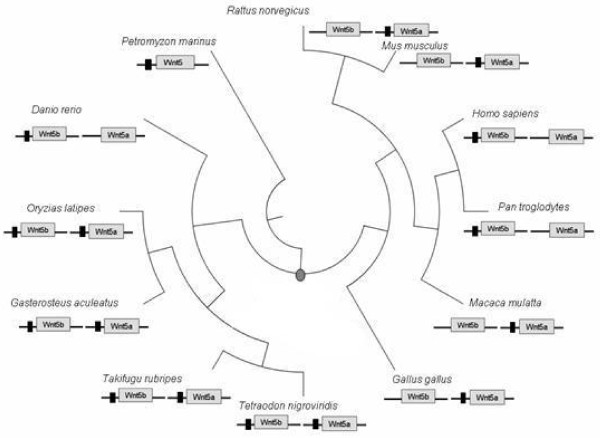
**Plylogenetic tree of the *taxa *included in the regulatory region analysis**. The circular phylogenetic tree shows the evolutionary relationships for those species, used for the regulatory region analysis. The putative time of the gene duplication event is shown with the grey dot, corresponding to the divergence time of gnathostomata. Under the name of each organism the *Wnt5 *paralogs are shown, with the regulatory module, when present, marked with a black box.

Finally, Figure 4 shows those species in which this regulatory region was identified in at least one *Wnt5 *paralog. Interestingly, while this module is conserved in both *Wnt5 *paralogs of gnathostomata fishes, for one representative of this class, *Danio rerio *(zebrafish), as for mammals and birds, it was identified in only one paralog (Figure 4). Concerning the regulatory module's loss in the *Wnt5 *of zebrafish, it has been shown that in teleosts after gene duplication only one gene maintains the regulatory module in its neighborhood [21].

### Functional Domain Analysis

In order to understand why both *Wnt5 *genes were preserved after the duplication event, whereas only one maintained the regulatory module, we conducted a functional domain analysis (see Material and Methods). Our analysis revealed the presence of the same functional domain at approximately the same position in both proteins. More precisely in mice, Wnt5a and Wnt5b, included the domain Wnt1 in the positions of their amino acid sequences, 51-360 and 63-372, respectively, while those of *D. rerio *in the positions, 65-374 and 54-363 (Data available upon request).

Wnt1 domain is known to be implicated at the development of multicellular organisms, the Wnt receptor signalling pathway, and the calcium modulating pathway. The canonical Wnt signalling pathway (Wnt/β catenin pathway) is involved in cell fate specification and proliferation [31] and the other two non canonical (b-catenin independent) Wnt signalling pathways are implicated into organ formation through antagonising the canonical Wnt signalling pathway [4,32]. In vertebrates, the latter ones regulate cell polarity and guide cell migration events during embryogenesis leading into endoderm patterning and more precisely in pancreas formation (see 33 and references therein).

A recent study [33] has shown that in zebrafish while Wnt5b activates the non canonical Wnt signalling pathway, contemporaneously acts as a negative feedback loop for the regulation of the canonical one. On the other hand, Topol et al., 2003 [32], showed that in mouse the inhibition of the canonical pathway is attributed to the *Wnt5a *gene. Summarizing, the conservation of both *Wnt5 *paralogs after the duplication event is related to a negative cross regulatory inhibition between the canonical and the non canonical Wnt signalling pathways, maintaining a responsive backup circuit [34]. From the above, we may suggest that in the pancreatic development the expression of mouse *Wnt5a *and zebrafish *Wnt5b *genes, both carrying the conserved regulatory module, in greater amounts than their paralogs is necessary, in order to inhibit the canonical Wnt signalling pathway and achieve a proper cell migration for the pancreatic islets formation.

### Expression Profile Analysis

Within this section, the expression pattern of the human and mouse *Wnt5a *and *Wnt5b *genes were investigated in several adult human tissues and during mouse embryonic development of pancreas. In the first case, expression patterns of human *Wnt5 *paralogs were retrieved from GeneCards V3 database [35] and from the publicly available data of Su et al., 2004, [36]. Both approaches revealed a similarity between human *Wnt5 *paralogs expression patterns (see Additional file 1, Panel A and B), supporting the previously referred hypothesis of genomic conservation related to a responsive backup circuit [34], in which both genes participate. Indeed, taking into account that during early somitogenesis, the endoderm patterning initially requires suppression of the canonical Wnt signalling, while later an increased Wnt/beta activity is necessary [37], we understand that both genes are implicated in the regulation of the canonical and non canonical Wnt signalling pathways.

On the other hand, the expression patterns of both mouse *Wnt5 *paralogs, as given from microarray experiments investigating the pancreatic development in early murine embryos from day 12.5 until 16.5, revealed distinct profiles in their expression (see Additional file 1, Panel C). More precisely, until 13.5 days of mice pancreatic development the expression level of *Wnt5a *gene (carrying the module) is higher to that of *Wnt5b*, while at 14.5 day, where pancreatic ducts become visible [15], both genes hold similar expression. Afterwards (until day 16.5), *Wnt5a *gene further decreases its expression, reaching lower levels than those of *Wnt5b*. Summarizing, the above described pattern indicates that as pancreatic development proceeds the amount of *Wnt5a *gene gradually decreases, while that of *Wnt5b *is maintained constant. In this case, as we have already described, the murine *Wnt5a *(carrier of the conserved regulatory module) gene, controls the proper migration of the cells in order to properly form the pancreatic islets [5]. This last procedure, is estimated to take place approximately during those days for which *Wnt5a *expression is higher to that of *Wnt5b *(see Additional file 1, Panel C). Indeed in early mice embryos of 11.5 days, the insulin precursors of mature b-cells appear [15] and afterwards they migrate along the ducts and blood vessels in a cord like linear pattern in order to form the islets (see 38 and references therein), where until the 14^th ^day they are arrayed as single cells within the ductal epithelium. Finally, at a 16 days old embryo, endocrine cells begin to organize into islet like clusters, and when the embryo reaches the 18^th^-19^th ^day, the islets are fully formed [39].

Summarizing, the expression patterns shown here for *Wnt5a *and *Wnt5b*, in human and mouse, represent additional evidence on why both genes are conserved after duplication, and indicate the functional role of this module in the *Wnt5a *gene during mice pancreatic development.

On the other hand, the shift of the regulatory module in rhesus monkey *Wnt5a *compared to human *Wnt5b*, prompted us to further investigate expression data of *Wnt5 *genes in embryonic stem cells and several adult tissues of Macaque. The obtained profiles in both analyses (stem cells and adult) showed that *Wnt5a *gene (carrier of the module) is constantly expressed, while *Wnt5b *not (Panels A, B and C of Additional file 2).These results indicate that the conservation of the regulatory module in a paralog is linked with a functional advantage.

## Conclusions

In this study, phylogenetic and regulatory region analyses of *Wnt5 *genes were conducted. A special feature of the phylogeny in our work is the inclusion of Lamprey, the only living representative of the agnatha (jawless), with known genome, from which all jawed vertebrates diverged [40]. The phylogenetic analysis confirmed that in those species arisen after the divergence time of gnathostomata two *Wnt5 *paralogs exist, whereas in the agnathon Petromyzon *(P. marinus) *and the representatives of *protostomia *(*D. melanogaster*) and *deuterostomia *(*C. intestinalis*) invertebrates, only one (see Figure 1). This is explained by a duplication event which has occurred at the evolutionarily time that gnathostomata diverged from agnatha (before 560 MYA; 1, 2).

Finally, the comparative regulatory region analysis allowed the identification of a conserved region, where many TFs, annotated to participate in pancreas formation, bind and regulate *Wnt5 *expression. The presence of these binding motifs, which cluster inside this regulatory region, might explain the implication of the corresponding *Wnt5 *paralog into the processes enlisted necessarily for the pancreatic cells subtypes' differentiation. Providing the fact that the *Wnt5 *gene is a member of the *Wnt *family, it is interesting to identify LEF1, a known mediator of Wnt signalling pathway [30], as a regulatory factor of *Wnt5 *expression, which further guides this pathway. On the other hand, the regulation of the *Wnt5 *gene through TFs with known implication in pancreas organogenesis, and more specifically in the proper orchestration of cell movements, gives evidence for the necessity of this regulatory module for the proper differentiation of cells capable of producing insulin. It is worth pointing out that, Lamprey with both pancreas and insulin producing cells has preserved this module inside the regulatory sequence of the *Wnt5 *gene.

Taking into account that the evolutionary time, when *Wnt5 *and its regulatory sequence were duplicated, is close to the divergence time of gnathostomata, it is not surprising that in some gnathostomata fishes this module was identified in both *Wnt5 *paralogs, while for evolutionary younger jawed vertebrates, together with zebrafish, it is preserved in only one. It is worth noting that in Osteichthyes, in which zebrafish belongs, regulatory module divergence and subfunctionalization between paralogous genes are common phenomena [41]. Evaluating the results of our study we may conclude that the *Wnt5 *paralog, the regulatory module of which was lost (*Wnt5*a of zebrafish and *Wnt5b *of mouse) was excluded from the participation of the pancreatic cells' subtype specification procedures. This could be characterized as a neofunctionalization rather than as a subfuntionalization event. Indeed, as we have already anticipated, a study of Kikuta et al., 2007 [21] has shown that in teleosts there are numerous cases, where a highly conserved regulatory module remains near to only one duplicated gene, providing a satisfactory explanation why in zebrafish this regulatory module was maintained only in the *Wnt5b*, which is also the one implicated in pancreas development [3].

The conservation of this regulatory module in at least one paralog of the species investigated in this study (Figure 4) reflects its functional importance. Indeed, the experimental data from mouse and zebrafish reinforce a possible relation between the presence of this regulatory module in a *Wnt5 *paralog, and its implication in pancreas formation [3,5]. Moreover, the presence of this TFBS cluster in human *Wnt5b*, absent in that of the rodents (mouse and rat), not only explains the observed human-rodents divergence of *Wnt5b *promoters [42], but also agrees with its implication in human type 2 diabetes [43]. Finally, the herein examined profiles for *Wnt5 *genes for two distinct cases; mice pancreatic development and across several human adult tissues add evidence on the functionality of this module and explain why genic preservation has occurred respectively. Indeed, the first case supports the fundamental role that mouse *Wnt5a *(carrier of the regulatory module) plays during pancreatic development. On the other hand, the human adult tissues expression profiles reveal a low expression divergence between *Wnt5 *genes in the adult state, which supports the preservation of *Wnt5 *genes, known to be implicated into a responsive backup circuit [34]. Concluding, it is very likely that selective pressure through evolution forced the coding regions of *Wnt5 *paralogs to remain unaltered and maintained the functionality of at least one regulatory module inside their upstream sequence. This last point is further supported by the expression profiles of macaque *Wnt5 *genes.

Despite the fact that some issues presented herein, might require further experimental exploration, the approach adopted in this work managed to offer a putative explanation why either *Wnt5a *or *Wnt5b *across different species preserved the same function. In relation to this, we conclude suggesting that similar analyses associated to experimentally produced data, could represent an efficient strategy in order to investigate cases where a shift in the functional role of paralogs has occurred.

## Materials and methods

### Ortholog Identification and Phylogenetic Analysis

*Wnt5a *and *Wnt5b *orthologs were identified with the Reciprocal Best Blast Hit method [44-46], using as reference sequences the murine Wnt5a and Wnt5b proteins, as retrieved and extracted from Ensembl database ([47]; Ensembl release 52-Dec 08, Ensembl release 53 - Mar 2009), with accession numbers, [Ensembl: ENSMUSP00000107891 and Ensembl: ENSMUSP00000032273] respectively.

Syntenic genes to *Wnt5 *paralogs were identified using the same version of Ensembl database in combination with the ortholog identification method [44-46]. Protein alignment of the orthologous peptides was performed using ClustalW [48]. On the basis of this alignment, and using Bioedit and Phylip package [49] a phylogenetic tree was reconstructed with the Maximun Parsimony method setting a bootstrap value of 2000 [50-52]. The commonly accepted tree of the *taxa *shown in Figure 4 was extracted from NCBI database, using the Taxonomy Browser [53]. Trees presented in this work were visualized using TreeExplorer in MEGA 4.0 [54].

### Functional Domain Analysis

The functional domain identification in both Wnt5 protein sequences was performed using the SMART algorithm [55] and the protein sequences of Wnt5a and Wnt5b for mouse and zebrafish as queries (mouse accession numbers [Ensembl: ENSMUSP00000064878 and Ensembl: ENSMUSP00000112448]; zebrafish accession numbers [Ensembl: ENSDARP00000018037 and Ensembl: ENSDARP00000041851]).

### Regulatory Region Analysis

The genomic regions of the orthologous *Wnt5a *and *Wnt5b *genes, spanning 4500 bp upstream to 500 bp downstream from the TSS were extracted from Ensembl database [47] for the species given in Figure 3. The TSS for every gene was annotated also at Ensembl Database [47]. The extracted regulatory regions were submitted to the MatInspector platform in Genomatix Database [56]. The putative TFBS for all TFs from Matrix Library 7.1 were detected using a core similarity value of 0.75, and the "optimized" matrix similarity as cut- off parameters. In order to increase robustness of our analysis, we sought only the common TFBS for the orthologous sequences of each gene. As a second filtering step we searched the sub-regions with maximum length of 1000 bp, which contained at least one binding site for the TFs Neurogenin1/3 (NGN1/3), clustered at the NEUR TF family. More precisely, only those NEUR TFBS with the highest core and matrix similarity values, inside the regulatory sequences of every species paralogs, were selected as anchors. This parameter was implemented since *Wnt5a *gene has been shown to be regulated by NGN3; a conclusion drawn by the fact that its expression levels were found to be altered after NGN3 induction [9]. Considering these NEUR TFBS as anchors and in combination with a methodology described in a previous work [18], we searched the TFBS of other common elements (TFs) in all orthologs from the species shown in Figure 3. In conclusion, we tried to discover regions where TFBS from TFs belonging to NEUR family were co-localized with those of other *trans *regulatory elements, also implicated into pancreas differentiation procedures.

The above analysis was repeated for *b-actin*, after we identified the orthologous genes (query sequence: murine *b-actin*; [Ensembl: ENSMUSG00000029580]) for the same number of species (Figure 3) covering the same phylogenetic distance to those of *Wnt5 *genes.

### Expression Profile Analysis

Expression patterns for both *Wnt5a *and *Wnt5b *genes in human, mouse and macaque were investigated. For the human *Wnt5 *paralogs, expression data were retrieved from two sources: the GeneCards V3 [35] and the dataset browser of GEO database (Gene Expression Omnibus; 57). More precisely, in the second source, expression data were retrieved and compared for the replicates of 79 physiological human tissues, provided by Su et al., 2004 [36].

Concerning the murine *Wnt5 *genes, the analysed expression data were also retrieved from the dataset browser of GEO database, [57] from microarray experiment during mice pancreatic development, using the Affymetrix Murine Genome U74 Version 2 array [58].

For the macaque, expression data were retrieved from embryonic stem cells [59], and from several adult tissues [60,61].

## Abbreviations

MYA: Million Years Ago; TF: Transcription Factor; NGN3: Neurogenin3; TFBS: Transcription Factor Binding Sites; TSS: Transcription Start Site; NGN1/3: Neurogenin 1/3.

## Competing interests

The authors declare that they have no competing interests.

## Authors' contributions

SK conceived the present study and designed the *in silico *approach. MK carried out the acquisition, analysis, interpretation of the data and drafted the manuscript. SA offered valuable insight to the interpretation of the results and conducted the expression profile analysis. All authors revised and approved the final version of the manuscript.

## Authors' information

MK: PhD Student in the Department of Pharmacy of University of Patras and in the Biomedical Research Foundation of the Academy of Athens (BRFAA).

SA: PhD, post-doc in Bioinformatics & Medical Informatics Team, in the BRFAA.

SK: DPhil, Investigator C-Assistant Professor Level, Head of Bioinformatics & Medical Informatics Team, in the BRFAA.

## Reviewers' comments

### First Reviewer

Ran Kafri (nominated by Yitzhak Pilpel)

Lahav Lab, Department of Systems Biology, Harvard Medical School

#### Report

I read your manuscript and found it very interesting. Gene duplications have always been close to my heart. It was very interesting that you were able to identify both synteny and regulatory motif conservation. This conservation indicates the importance of the functional role played by Wnt and of the lack of redundancy in the time of duplication. I think that the main aspect that may improve the work is to embed it within the larger framework of current models describing duplicate gene evolution. Specifically, the fate of duplicate genes, following a duplication event, is typically described in terms of one of three possibilities (see ref 1). Neofunctionalization, subfunctionalization and gene loss. Obviously, this above description is a form of simplification. For example, a pair of duplicates can undergo both partial subfunctionalization and neofunctionlization. Nevertheless, the ne-/sub funcitonalization model serves a very good axis for describing fates of gene duplicates. In the case of Wnt5, which of the two processes would you say is a better descriptor of what happened?

Another meta-question that is interesting to address is why, following the gene duplication event, have both Wnt5a and Wnt5b been evolutionarily conserved? Redundancy is known to be evolutionarily instable. The fact that both duplicates have been retained throughout evolution suggests that there is a functional advantage to having both (see ref 2). What is that advantage? Maybe a guess at that question could be based on the motif analysis of both genes. Which motifs are old, which are new. What processes do these below to?

ref 1:

The evolutionary fate and consequences of duplicate genes. Lynch M, Conery JS. Science. 2000 Nov 10;290(5494):1151-5.

ref 2:

The regulatory utilization of genetic redundancy through responsive backup circuits. Kafri R, Levy M, Pilpel Y. Proc Natl Acad Sci USA. 2006 Aug 1;103[31]:11653-8. Epub 2006 Jul 21.

##### Authors' response

We do agree with reviewers' comments, and we have now added in our manuscript a possible model describing Wnt5 gene duplication event, and on why both copies were preserved. We hope now to fully answer to the comments of the reviewer.

### Second Reviewer

Sarath Chandra Janga (nominated by Sarah Teichmann).

MRC-Laboratory of Molecular Biology. Darwin college, University of Cambridge.

#### Report 1

In this work the authors present an evolutionary and regulatory region analysis for the Wnt5 family of genes in different vertebrate and invertebrate genomes. Although the work is interesting and in general well-presented, I have few concerns regarding the presentation and clarity of the manuscript. Given that the manuscripts only major observations are that Wnt5 gene is duplicated early in evolution after the divergence time of gnathostomata and that in several genomes with paralogs the regulatory region corresponding to wnt5a and wnt5b paralogs appears to be conserved, the authors should consider addressing all these comments below in order to increase the readability and clarity of the manuscript.

##### General Comment 1

The title appears very abstract without any novel implications although the authors did carry out some meticulous first hand analysis. So I suggest changing the title to something more informative like "Phylogenetic and regulatory region analysis of Wnt5 genes reveals change in genomic context of the two paralogs [despite conservation of regulatory elements]"

##### General Comment 2

One major concern with the study is that the authors have a series of observations with regard to the regulatory region analysis but it is not evident if what are the interpretations of these observations, as the authors never conclusively discuss them in the context of known knowledge about Wnt signalling. For instance, the authors mention very briefly an show in Figure 3 that only one of the two Wnt5 paralogs contain the regulatory module in several higher eukaryotes however it is not clear if this is due to sensitivity of the search method adopted or if this is true what are the implications in terms of the expression patterns of the other Wnt gene. These observations should be discussed in detail.

##### General Comment 3a

In this context, more generally how do the expression levels of wnt genes in various human and mouse tissues compare? For instance using the microarray datasets like Andrew Su et. al. PNAS for expression data do the authors see a correlation in their levels or a divergence.

##### General Comment 3b

It appears to me that the authors claim (and expect) at some point in the manuscript that they are both still involved in the same tissue-specific processes (these observations primarily based on previous expression studies) however the lack of regulatory module conservation with change in neighborhood suggests the opposite- So the authors should discuss their observations in light recent studies such as Janga et. al. PNAS 2008 -and De et. al. Genome Research 2008 where the authors show rewiring of regulatory networks due to change in context.

##### General Comment 4

The authors show in Figure 3 that NGN3 binds to wnt5b in both human and chimp but not wnt5a however the discuss in the introduction in light of references 7 and 8 that wnt5b expression is unaltered while wnt5a changes in expression due to the induction of NGN3. This has to be clarified in light of authors (apparently) contrasting observations.

##### General Comment 5

The authors should consider introducing/showing the phylogeny shown in Figure 1 in the introduction itself and mark the genomes into different groups of vertebrate and invertebrates on the figure to improve clarify. Do the authors see the same tree if the alignment was done by grouping wnt5a and wnt5b proteins as different groups.

##### General Comment 6

In most locations of the manuscript, the authors do not cite corresponding materials and methods section.

##### General Comment 7

What do the authors mean by ".. between the evolutionary age of *P. marinus *and that of gnathostomata" towards the end of the section on phylogenetic analysis?

##### General Comment 8

How do the author identify the Transcription Start Site (TSS) in various genomes? are these a mix of experimental and computational predictions. This has to be detailed in the methods.

##### General Comment 9

Related to point 8 above the authors could use the relative locations of TSS and the binding regions of TFs to propose whether the regulatory context is indeed conserved between the paralogs across genomes analyzed.

##### General Comment 10a

The authors should discuss the following scenarios based on their findings What is the likelihood that the paralogs for which there is conservation of regulatory module there is lack of expression/process context.

##### General Comment 10b

Those paralogs which don't have the regulatory module are they expressed in general? or alternatively is it possible that they are non-functional over times.

###### Minor comments for revision

There are grammatical inconsistencies and lack of clarity in presentation in different parts of the manuscript, which I expect the authors to revise. Below is an incomplete list which should be corrected.

1) Introduction, Rephrase the sentence to improve clarity ".. and ciona intestinalis (deuterostome), and to early vertebrates, like agnatha, including hagfish and lampreys, were."

2) Rephrase the sentence in Phylogenetic analysis section "The orthologous peptides for Wnt5a and for their wnt5b paralogs were identified.."

3) Page 4, "Formaly" should be "Formally"

4) In conclusions section rephrase ".., situated in close proximity one to each other, and.."

5) On page 10, para 2 change "evolutionary" to "evolutionarily"

6) Rephrase Page 11, last para, "..on why for some species different Wnt5 paralogs

7) The authors use regulatory element and module synonymously in different places. Since the search was for a set of regulatory elements, they should use regulatory module or set of associated TFBS as a common terminology throughout the manuscript in order to avoid confusion to non-specialists.

#### Authors' response

##### Answer to General Comment 1

Having taking into account the constructive suggestion concerning the title of our manuscript, we changed it to read: "Phylogenetic and regulatory region analysis of Wnt5 genes reveals conservation of a regulatory module with putative implication in pancreas development."

##### Answer to General Comment 2

We agree with this comment of the reviewer. In order to test whether the presence of this module in Wnt5 genes is due to sensitivity of our method, we repeated exactly the same analysis (same parameters and for the same species) for the constitutively expressed gene of b-actin, finding absence of the module, with exception that of zebrafish. For details please see section Regulatory Region Analysis.

##### Answer to General Comment 3a

We agree that an estimation of a correlation in expression divergence of Wnt genes in various human and mouse tissues would be a very interesting topic. However, we believe that it would alter the main scope of our work, which is focused on investigating the implication of Wnt5 genes in pancreas development, based in the background knowledge that in mice embryonic stem cells upon Ngn3 induction (key regulator of pancreas development) Wnt5a gene shows the greatest alteration in expression compared to the other Wnt genes, i.e., Wnt4 and Wnt11 (Serafimidis et.al., 2008). Moreover, we believe that the suggested analyses by the reviewer, involving the expression divergence of several Wnt genes for only two species (mouse and human; Su et al., 2002, 2004) in adult state, would not add new elements on our study which is limited to the Wnt5 paralogs of several species during embryonic pancreas development.

##### Answer to General Comment 3b

Our manuscript suggests that only the Wnt5 paralog carrying the regulatory module is implicated into the endocrine pancreas formation, and not both. However, those Wnt5 genes without the element have important role in other processes and in light of some additional analyses we now explain why both copies were conserved (see new section Functional Domain Analysis). Finally, concerning the studies of Janga et al., (2008) and De et al., (2008) both reporting important observations at genomic level, in our opinion, it cannot be directly related to our work, where the paralogs of a single gene (Wnt5) across several species is investigated, and not several genes within an organism.

##### Answer to General Comment 4

Reference 7 and 8 are not contrasting to what is shown in Figure 3 for human and chimp, since these studies refer to murine cells.

##### Answer to General Comment 5

We now refer Figure 1 also in the introduction, and we added vertebrate and invertebrates indication.

We repeated the analysis with Wnt5a and Wnt5b proteins separated and results were maintained rather constant to the submitted tree (details on the results are available upon request).

##### Answer to General Comment 6

We have now reviewed the manuscript and where necessary it has been added (see materials and methods).

##### Answer to General Comment 7

We have now rephrased this section of our manuscript.

##### Answer to General Comment 8

We now added the above in the material and methods section

##### Answer to General Comment 9

We have now added a comment on the relative positions of TSS and the binding regions of the TFs.

##### Answer to General Comment 10a

In the two last paragraphs of our manuscript we clearly report that our conclusions are limited only to those organisms where experimental data support the relation between conserved modules and function, the latter one additionally supported by the results of the suggested negative control experiment. In our opinion any further discussion concerning the presence of the module and lack of its expression/process, would be purely speculative in absence of any experimental information or evidence.

##### Answer to General Comment 10b

As we already anticipated, our study is focused on those Wnt5 genes implicated in pancreas developmental processes guided by Ngn3 induction. However, it has already been shown that both, Wnt5a and Wnt5b paralogs, are necessary for several developmental processes demanding proper cell migration and cell polarisation events (Cooper et. al., 2006, Hardy et al., 2008, Yang et al., 2003, Kim et al,, 2005). In this regard we have now included in our work a comment on their implication in different processes, and on why both Wnt5 genes were preserved.

Answer to the Minor comments for revision

We have corrected all minor comments.

#### Report 2

Thanks for addressing some of the concerns raised by me however i still feel that the manuscript can be significantly strengthened by improving two aspects of the study

1) By improving the discussion in light of your observations by extrapolating to observations made by other groups in various tissues

2) I still feel that since the manuscript is already a very short piece (as it is) and so it warrants an in depth analysis of expression patterns at least in pancreas and related tissues if not across all the tissues for which expression data is available.

This analysis is rather easy using currently available gene centric databases such as 'genecards' for humans and i urge the authors to do this analysis and present a heatmap showing this to support the discussion part. If you are able to address these minor concerns I am willing to support the publication of your work.

#### Authors' response

According to your suggestions, we investigated the expression patterns of both Wnt5 genes, in several adult tissues of human and during pancreatic development of mice.

For the human Wnt5 genes, a similarity in their expression profiles was observed (for details please refer to the main text of the updated version of our manuscript, attached in this email). The high correlation in their expression profiles in adult state of human is in agreement with the conservation of both genes in order to maintain a responsive backup circuit, which controls the canonical and non canonical Wnt signaling pathways.

Concerning the expression pattern of the murine Wnt5 genes during pancreatic development, we found a distinct and well maintained profile in their expression. More precisely, during those days of pancreatic development in which the migration of those cells forming the pancreatic islets is estimated to take place, the murine Wnt5a gene (carrier of the conserved regulatory module) shows a higher expression than Wnt5b. Summarizing, the given results and observations, from the herein conducted expression analyses, agree with what we describe in the manuscript (please see attached manuscript for details).

We look forward to hearing from you and thanking you very much for your constructive comments and the time you took to review our manuscript thoroughly.

#### Report 3

Thank you very much for addressing the concerns which I raised in my previous round of comments. The manuscript now reads well and I think is suitable for publication.

### Third Reviewer

Andrey A. Mironov (nominated by Mikhail Gelfand).

Department of Bioengineering and Bioinformatics, Moscow State University.

#### Report 1

The paper gives some observations about evolution and regulation of Wnt5 genes. On my opinion the paper has very limited interest. Found some paralogs in some genomes, found some binding sites. So what?

Genome contains about 20k more or less conserved genes. The same work can be done almost for every gene in genome with almost the same result � some paralogs, some regulation...

At least the analysis of regulation should be extended to find co-regulated genes and to reconstruct a genetic network.

Some particular comments:

Comment 1

The direction of Wnt5 genes on fig. 2 should the same on all lines. For example the line 2 on fig 2a should be presented on opposite direction.

Comment 2

The analysis of regulatory modules should be given not only for species with insulin producing cells. Structure of possible modules for other organisms can be used as a control and will be able to show some possible evolution features of the system.

#### Authors' response

In a previous work (Kapasa et al., 2008) has been proposed a putative regulatory genetic network which leads into the differentiation of the endocrine pancreas cell sub-types. Taking into account that the extension of a similar analysis upon Wnt5 genes would not change the previous model, we decided to focus our work on the relation between the conservation of the regulatory module and its functionality in pancreas development, as supported by experimental data.

##### Answer to Comment 1

In Figure 2 we decided to maintain the relative direction of the syntenic genes as given by Ensembl database (5-3 or 3-5), in order to help the reader understand the modules' position, and provide information concerning the conservation of the orientation of the genes in the investigated species.

##### Answer to Comment 2

Towards this direction, we have now performed a negative control experiment using b-actin orthologs covering the same phylogenetic distance, and including the same number of species to those for Wnt5 genes. This analysis showed absence of regulatory module, with the only exception the b-actin of Zebrafish, in which a cluster of the same TFBS was identified, in a region of approximately the double length to that of the Wnt5 in zebrafish. Moreover, for the Wnt5 genes of those organisms without pancreas and insulin producing cells (Ciona and Droshophyla) we did not found the Ngn1/3 binding sites, which is the main criterion for the identification of the regulatory modules.

#### Report 2

I do not change my opinion now. In current form the paper have limited interest. It gives some information but not knowledge.

##### Comment 1

A negative control on b-globin is useful. But to understand the quality of prediction of regulatory modules you should provide real control - to search such modules for about 1000 random selected genes.

##### Comment 2

Other way is to provide search such regulatory module genome-wide (In cited paper only some other Wnt genes were analysed) to find possible coregulated genes and to analyse consistency of set of coregulated genes in different genomes.

##### Comment 3

Fig 2: The direction of Wnt5 genes should be the same (as it is done in, for example, Strings database). In this case the evolution events of gene rearrangement will be clear. The gene orientation in genome databases is more or less random and it is not related to evolution events.

##### Comment 4

Fig 4: We can see something interesting: in human and chimp the regulatory module exists in upstream region of Wnt5b while very close related organism - macaque - has such module in upstream of Wnt5a. Seems the regulation is switched from one gene to another. Or may be the filter is too strong and both genes really are regulated but the PREDICTED regulatory efficiency are slightly different?

#### Authors' response

##### Answer to Comment 1

Following your suggestion, we used the non-expressed in embryonic stem cells gene of b-globin, as a second negative control marker. We found absence of the regulatory module, as for actin genes (first negative control marker). The results from the negative control markers, combined with those coming from the additional analyses of Wnt5 paralogs expression profiles in several human adult tissues and during mouse embryonic development (See additional file 2 of the updated version of the manuscript), reinforce not only the functionality of this conserved module in Wnt5 genes, but also the validity of our method.

"*But to understand the quality of prediction... 1000 random selected genes*."

We believe that such an analysis would be justified if our study was a genome-wide study, and not a study restricted to a specific gene (Wnt5) during a specific process (pancreas development).

##### Answer to Comment 2

Our study is limited to the paralogs of Wnt5 gene in several species, in order to give a putative explanation for the shifting role concerning pancreas development among these genes. However, a similar regulatory region analysis in experimentally verified co-regulated genes (WIPS1 and CTGF) has been applied elsewhere with satisfactory results (see Kapasa et. al., 2008).

##### Answer to Comment 3

We still do not find necessary the modification of Fig. 2. In our opinion, the availability of the genes' relative orientation allows the reader to correlate the genomic translocations among organisms, when present, with the preservation of the regulatory module always in the 5' region of the gene in every case. In addition, several studies preferred showing the orientation of genes, as we did in figure 2 (Kawahara and Lambeth, *BMC Evolutionary Biology 2007, 7:178; *Jane et. al., *Natur*e *2002*, *419: 512-519*, *Roach et al., Proc Natl Acad Sci USA. 2005, 102: 9577-9582*).

##### Answer to Comment 4

Concerning this point, we performed an additional investigation. Strikingly, for the Wnt5 genes of Rhesus Monkey, we extracted expression data from embryonic stem cells http://www.ncbi.nlm.nih.gov/geo/query/acc.cgi?acc=GSE4446 and from several adult tissues (http://www.ncbi.nlm.nih.gov/geo/query/acc.cgi?acc=GSE9531, and http://blast.wip.ncbi.nlm.nih.gov/geo/query/acc.cgi?acc=GSE7094), in order to investigate their expression profiles. Our results show that in the embryonic cells and the adult organs the Wnt5a gene (carrier of the module) is constantly expressed, while Wnt5b not (Panels A, B and C of Additional file 2). This different profiles between the macaque Wnt5a (module carrier) and Wnt5b (non carrier), the former being permanently expressed, suggest a functional advantage for the observed shift of the module's preservation within the macaque paralogs.

Besides that, we have already tested the sensitivity of our method in order to identify only functional regulatory modules either in both or in only one paralog of Wnt5 gene (see figure 4).

We thank you for your constructive criticisms, and we hope through our additional analyses to have fully satisfied your remarks concerning our study.

#### Report 3

Additional information about expression profile analysis is helpful.

But I can not find the Additional File 1 (seems you mean Table 1 in figure file?).

Now I have two suggestion.

First - to change fig.2! You are speculating about sintheny region (alignment of genes) but you do not want to show the information in aligned form! Why?

Second - some speculation about contradiction of phylogeny of Wnt5a and standard evolution tree needed. This contradiction is related to structure of regulation on fig 4. Seems here we can observe some positive selection events (have you checked it?) or/and some significant aminoacid changes. On my point of view this is the most interesting result.

By now I think you can publish the paper in current form (with changed fig 2 and fixed misprint with Additional File 1).

## Supplementary Material

Additional file 1 **Panel A: Human *Wnt5a and Wnt5b *expression profiles in healthy adult tissues**. **Panel B: **Graphical comparative representation of *Wnt5a *and *Wnt5b *expression profiles in healthy adult human tissues. **Panel C: **Graphical representation of *Wnt5a *and *Wnt5b *expression profiles in early mouse embryos. Click here for file

Additional file 2 **Panel A: Macaque *Wnt5a and Wnt5b *expression profiles in embryonic stem cells**. **Panel B and C: **Macaque *Wnt5a *and *Wnt5b *expression profiles adult tissues. Click here for file
