# Supplementary material for: Phylogenetic and regulatory region analysis of Wnt5 genes reveals conservation of a regulatory module with putative implication in pancreas development
Source: Biol Direct. 2010 Aug 4;5:49. doi: 10.1186/1745-6150-5-49 (PMC2922100; doi:10.1186/1745-6150-5-49)
Supplement: Additional file 1 — Panel A: Human Wnt5a and Wnt5b expression profiles in healthy adult tissues. Panel B: Graphical comparative representation of Wnt5a and Wnt5b expression profiles in healthy adult human tissues. Panel C: Graphical representation of Wnt5a and Wnt5b expression profiles in early mouse embryos. [file 1745-6150-5-49-S1.PPT]

## Slide 1
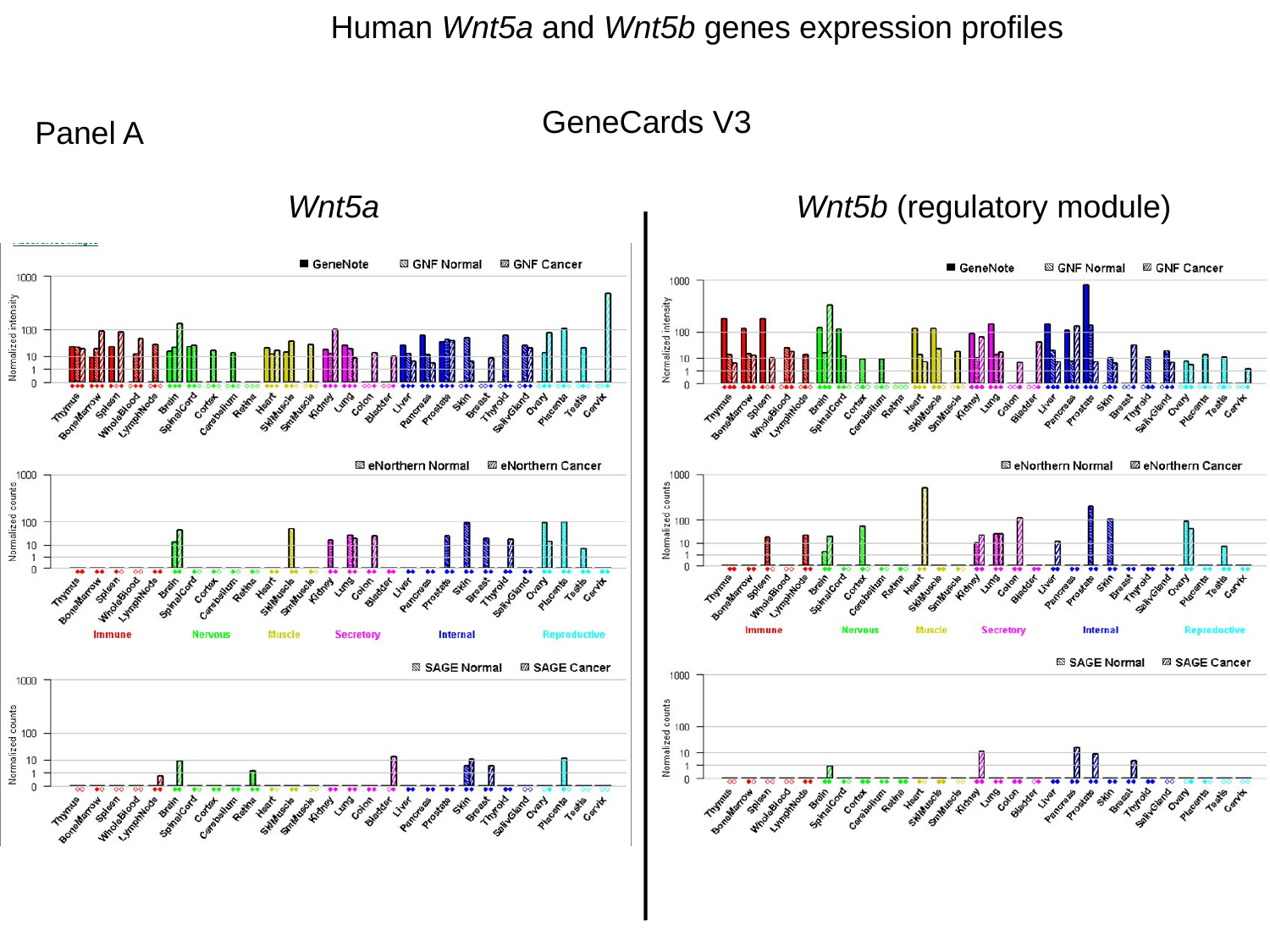

Human Wnt5a and Wnt5b genes expression profiles
GeneCards V3
Panel A
Wnt5a
Wnt5b (regulatory module)

## Slide 2
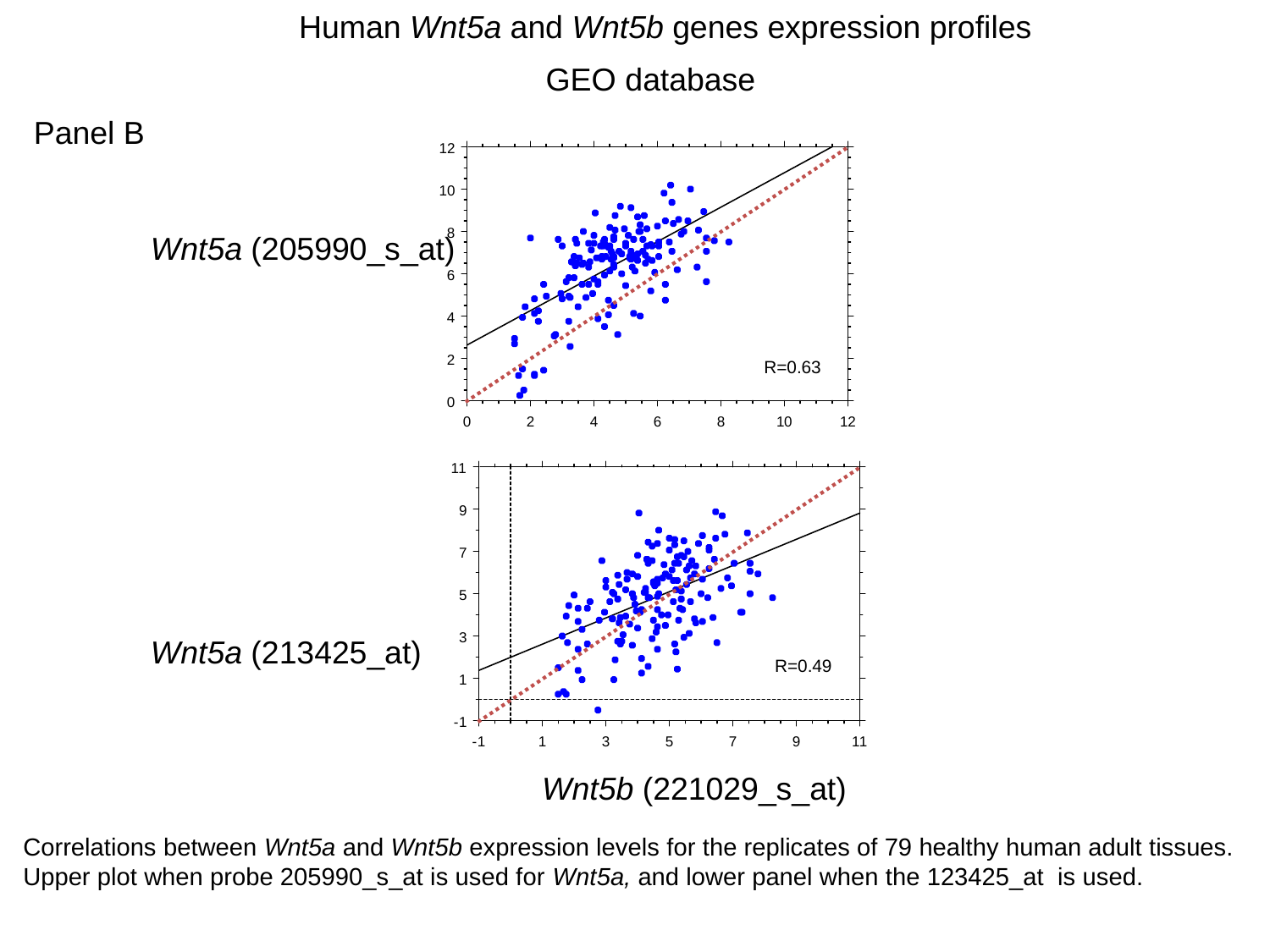

Human Wnt5a and Wnt5b genes expression profiles
GEO database
Panel B
Wnt5a (205990_s_at)
R=0.63
Wnt5a (213425_at)
R=0.49
Wnt5b (221029_s_at)
Correlations between Wnt5a and Wnt5b expression levels for the replicates of 79 healthy human adult tissues. Upper plot when probe 205990_s_at is used for Wnt5a, and lower panel when the 123425_at is used.

## Slide 3
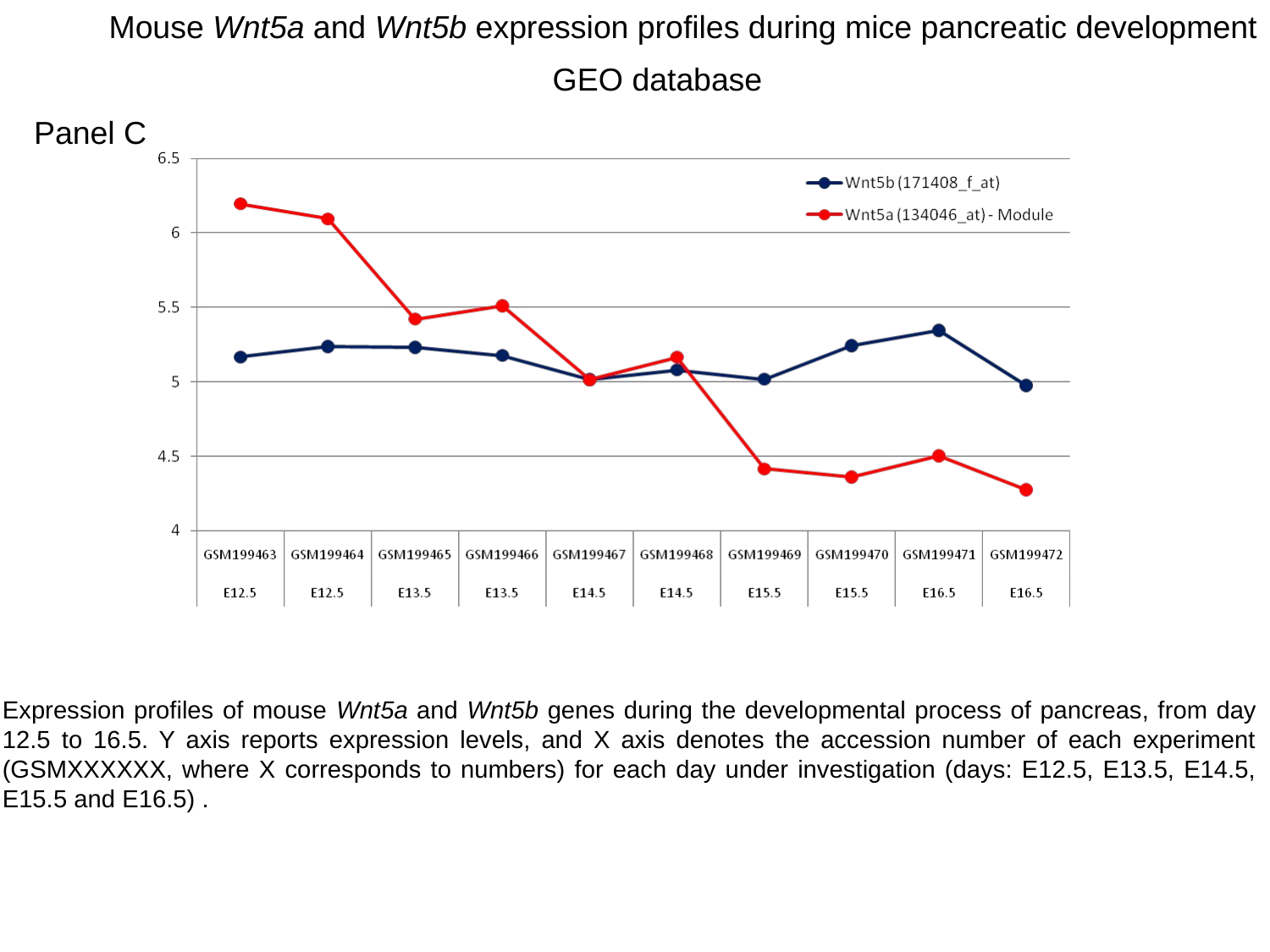

Mouse Wnt5a and Wnt5b expression profiles during mice pancreatic development
GEO database
Panel C
Expression profiles of mouse Wnt5a and Wnt5b genes during the developmental process of pancreas, from day 12.5 to 16.5. Y axis reports expression levels, and X axis denotes the accession number of each experiment (GSMXXXXXX, where X corresponds to numbers) for each day under investigation (days: E12.5, E13.5, E14.5, E15.5 and E16.5) .
