# Supplementary figures and images for: Phylogenetic and regulatory region analysis of Wnt5 genes reveals conservation of a regulatory module with putative implication in pancreas development
Source: Biol Direct. 2010 Aug 4;5:49. doi: 10.1186/1745-6150-5-49 (PMC2922100; doi:10.1186/1745-6150-5-49)

## Slide 1
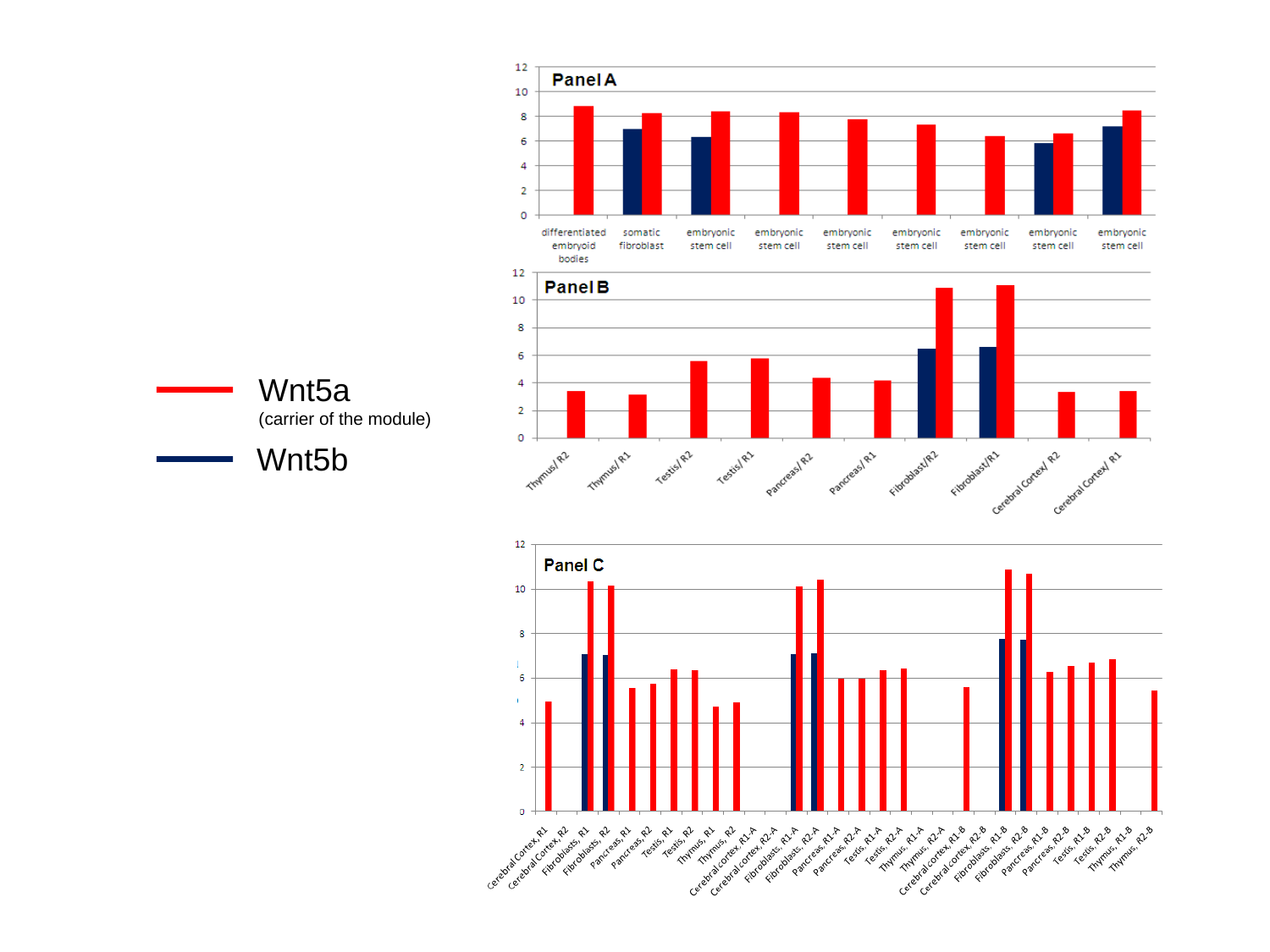

Wnt5a
(carrier of the module)
Wnt5b

Supplement: Additional file 2 — Panel A: Macaque Wnt5a and Wnt5b expression profiles in embryonic stem cells. Panel B and C: Macaque Wnt5a and Wnt5b expression profiles adult tissues. [file 1745-6150-5-49-S2.PPT]
